# S256. A META-ANALYSIS OF RECOVERY EDUCATIONAL AND AWARENESS INTERVENTIONS FOR MENTAL HEALTH PROFESSIONALS

**DOI:** 10.1093/schbul/sby018.1043

**Published:** 2018-04-01

**Authors:** Helena García-Mieres, Francisco José Eiroa-Orosa

**Affiliations:** 1 Universitat de Barcelona; 2 Universitat de Barcelona & Yale School of Medicine

## Abstract

**Background:**

The history of mental health care has been marked by various struggles for the dignity of service users. Some reform movements have started to use strategies aimed at professionals’ beliefs and attitudes change. This conference paper intends to systematically review and synthesize all information related to awareness-raising and training of professionals in aspects related to empowerment, recovery and in general in rights-based care to achieve full citizenship of mental health services users.

**Methods:**

We searched academic databases as well as web search engines, aiming at finding grey literature on the subject. Quantitative studies were included if they included mental health professionals, defined as all staff involved in the management of mental health service users, as well as mental health students. All participants included should have assisted to a recovery or psychosocial rehabilitation educational or awareness-raising program. Effect size of change in knowledge, attitudes and intention to implement recovery-based practice were meta-analyzed using a fixed effects model.

**Results:**

After a preliminary search, a total of 800 articles were added to a global database, of which 50 include explicit information on concrete trainings. Of these, 25 reported information about evaluation of the effectiveness of these training activities. Finally, 13 studies were included in the analysis, with a total sample size of 1123. Six studies adopted a repeated measures design and seven an independent group design (including RCTs and quasi-experimental studies). Recovery and rehabilitation based interventions had, on average, a small-to-medium-sized effect on knowledge of recovery principles (d+ = 0.33, 95% CI: 0.13 to 0.49); a small-to-medium-sized effect on attitudes to recovery principles (d+ = 0.36, 95% CI: 0.25 to 0.46), and a small-to-medium-sized effect on intention to implement recovery practice (d+ = 0.37, 95% CI: 0.02 to 0.71).

**Discussion:**

The results show positive effects of educational and awareness activities for mental health professionals. Elements such as duration and intensity of activities must be considered when analysing the persistence and applicability of the effects. More quality studies are needed to establish the active ingredients of these activities.

